# Dysfunctional oxidative phosphorylation makes malignant melanoma cells addicted to glycolysis driven by the ^V600E^BRAF oncogene

**DOI:** 10.18632/oncotarget.965

**Published:** 2013-04-08

**Authors:** Arnaldur Hall, Kathrine Damm Meyle, Marina Krarup Lange, Martin Klima, May Sanderhoff, Christina Dahl, Cecilie Abildgaard, Katrine Thorup, Seyed Moein Moghimi, Per Bo Jensen, Jiri Bartek, Per Guldberg, Claus Christensen

**Affiliations:** ^1^ Genome Integrity Unit, Danish Cancer Society Research Center, Denmark; ^2^ Centre for Pharmaceutical Nanotechnology and Nanotoxicology, University of Copenhagen, Denmark; ^3^ Research Unit for Dietary Studies Institute of Preventive Medicine Bispebjerg and Frederiksberg Hospitals, Denmark; ^4^ Seahorse Bioscience Europa, Copenhagen, Denmark; ^5^ Diet, Genes and Environment Unit, Danish Cancer Society Research Center, Denmark

**Keywords:** Oncogene addiction, melanoma, ^V600E^BRAF, the Warburg effect, glycolysis, oxidative phosphorylation

## Abstract

Oncogene addiction describes how cancer cells exhibit dependence on single oncogenes to escape apoptosis and senescence. While oncogene addiction constitutes the basis for new cancer treatment strategies targeting individual kinases and pathways activated by oncogenic mutations, the biochemical basis for this addiction is largely unknown. Here we provide evidence for a metabolic rationale behind the addiction to ^V600E^BRAF in two malignant melanoma cell lines. Both cell lines display a striking addiction to glycolysis due to underlying dysfunction of oxidative phosphorylation (OXPHOS). Notably, even minor reductions in glycolytic activity lead to increased OXPHOS activity (reversed Warburg effect), however the mitochondria are unable to sustain ATP production. We show that ^V600E^BRAF upholds the activity of glycolysis and therefore the addiction to glycolysis de facto becomes an addiction to ^V600E^BRAF. Finally, the senescence response associated with inhibition of ^V600E^BRAF is rescued by overexpression of glyceraldehyde-3-phosphate dehydrogenase (GAPDH), providing direct evidence that oncogene addiction rests on a metabolic foundation.

## INTRODUCTION

Oncogene addiction refers to a phenomenon that describes how cancer cells remain dependent on the constitutive activity of single oncogenes despite the acquisition of numerous genetic changes affecting both tumor suppressor genes and additional oncogenes [1]. When deprived of particular oncogenes, tumors have been shown to undergo almost complete regression whereas single cancer cells may undergo apoptosis or irreversible growth arrest i.e. senescence. For example, oncogene addiction has been demonstrated with respect to *MYC* in the context of multiple myeloma [2], hepatocellular carcinoma [3], osteosarcoma [4], and lymphomas [5]. Furthermore, addiction to the *HRAS* or *BCR-ABL* oncogenes has been observed in mouse models of melanoma and leukemia, respectively [6,7]. From a classical point of view, removal of single oncogenes from full blown cancers would not be expected to result in apoptosis or senescence since cancers uniformly harbor mutations in tumor suppressor pathways that inactivate normal control of cellular lifespan and apoptosis. At present, oncogene addiction constitutes the rationale behind newly developed treatment regimens targeting individual oncoproteins [8], but despite the proven clinical relevance little is known of the underlying causes for oncogene addiction.

The RAS-RAF-MEK-ERK signaling cascade plays a critical role in the transmission of signals to regulate gene expression and cell proliferation [9,10]. It is well established that the cascade is hyper-active in various types of cancer cells [10]. A broad range of human tumors have been found to contain *BRAF* mutations, including approximately 50% of melanomas [11]. Notably, one specific mutation, p.V600E/c.1799T>A, accounts for around 90% of *BRAF* mutations found in melanoma [12]. This mutation introduces a phosphomimetic conformational change in the activation domain of BRAF, which results in constitutive activation of the protein with a large increase in the basal kinase activity [13]. ^V600E^BRAF is an archetypical oncogene capable of transforming both fibroblasts and melanocytes [12,14]. Furthermore, cancer cells harboring ^V600E^BRAF exhibit oncogene addiction. Hence, RNA interference mediated knock-down of ^V600E^BRAF leads to senescence or apoptosis [15-17], and more recently, the BRAF inhibitors PLX4720 and its analog for clinical use vemurafenib (also known as PLX4032 or RG7204) were shown to inhibit ^V600E^BRAF with high affinity, causing cell death to melanoma cell lines *in vitro*[18,19], regression of metastatic melanoma in preclinical models [20] and prolonged survival of melanoma patients [21].

The addiction of melanomas to ^V600E^BRAF appears to reflect a more general addiction to the RAS-BRAF-MEK-ERK pathway, as MEK inhibitors CI 1040, U0126, AZD6244 and trametinib also cause cell death [22-24]. Notably, *RAS* and *BRAF* mutations predict sensitivity to MEK inhibition [24,25], and patients with melanoma harboring ^V600E^BRAF show comparable results with respect to tumor regression when treated with trametinib and vemurafenib [26]. Furthermore, the addiction to the core signaling cascade RAS-BRAF-MEK-ERK may extend and implicate upstream growth factors and their cognate receptor tyrosine kinases. We have previously shown that growth factors rescue malignant melanoma cells from senescence and apoptosis induced by knockdown of ^V600E^BRAF, providing that cells were still expressing ^WT^BRAF to allow signal transduction through MEK-ERK [17]. Later, growth factors and receptor tyrosine kinases have been shown to play crucial roles in acquired resistance to treatment with vemurafenib [27,28] confirming the functional overlap between exogenous growth factors and ^V600E^BRAF activity in terms of both mitogenic stimulation and survival.

Most cancer cells display a strikingly different metabolism than normal cells. The particularities of cancer metabolism are generated by various extrinsic factors such as pH and the scarcity of oxygen, glucose and other nutrients as well as cell-intrinsic mechanisms such as signaling pathways activated by oncogenes or controlled by p53, AMPK and mTOR [29-32]. The most prominent alteration, known as the “Warburg effect”, was first described by Otto Warburg in 1926. It describes how cancer cells continue to convert glucose to lactic acid through high rates of glycolysis even in the presence of abundant oxygen [33]. RAS, MYC and HIF-1α oncogenes are known to affect the expression levels of enzymes participating in glycolysis, the pentose phosphate pathway (PPP) and glutamine metabolism [34,35]. Both MYC and HIF-1α are direct targets of the RAS-BRAF-MEK-ERK pathway [36,37], suggesting an overall role of this pathway in the formation of various metabolic traits of cancer, including the “Warburg effect”. PLX4032 was recently demonstrated to reduce the uptake of 2(18F)fluoro-2-deoxy-D-glucose (18F-FDG) in melanoma cells, whereas inducible expression of ^V600E^BRAF increased the glucose uptake rate and decreased O_2_ consumption in thyroid cancer cells [38,39]. However, no evidence exists that ^V600E^BRAF regulates the expression of individual glycolytic enzymes, as seen with RAS and MYC. Also, potential bioenergetic factors responsible for the addiction of melanoma cells to ^V600E^BRAF remain unaddressed.

Here we provide the first evidence that ^V600E^BRAF causes the upregulation of genes involved in glycolysis, whereas knock-down of ^V600E^BRAF in melanoma cells conversely reduces the expression of these genes, lowers the rate of glycolysis and causes the reversal of the Warburg phenotype. Notably, using the recently developed Seahorse XF technology, we show two cases of ^V600E^BRAF-positive melanoma cell lines that contain dyscoupled mitochondria, rendering these cells dependent on ^V600E^BRAF-driven glycolysis for efficient ATP production. This work indicates that dysfunctional OXPHOS could be a factor contributing to oncogene addiction.

## RESULTS

### Melanoma cells contain active yet highly dyscoupled mitochondria

**Figure 1:**
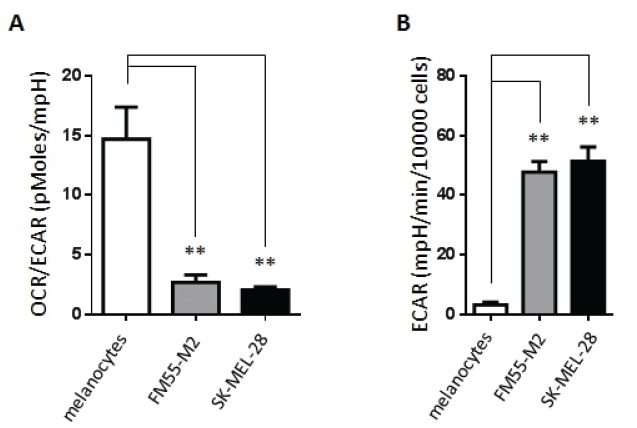
Basic metabolic characteristics of primary melanocytes and malignant melanoma cells (A) Ratio between oxygen consumption rate (OCR) and the extracellular acidification rate (ECAR) in melanocytes and melanoma cells. (B) Lactate production (ECAR, mpH/min/10000 cells) in melanocytes and melanoma cells (FM55-M2 and SK-MEL-28). Data are presented as the means ± SD (n = 12) from one experiment and are representative of three independent experiments. Statistical analysis were performed with one-way ANOVA using Tuckey's multiple comparisons test to calculate significance (** = *p* < 0.01).

**Figure 2:**
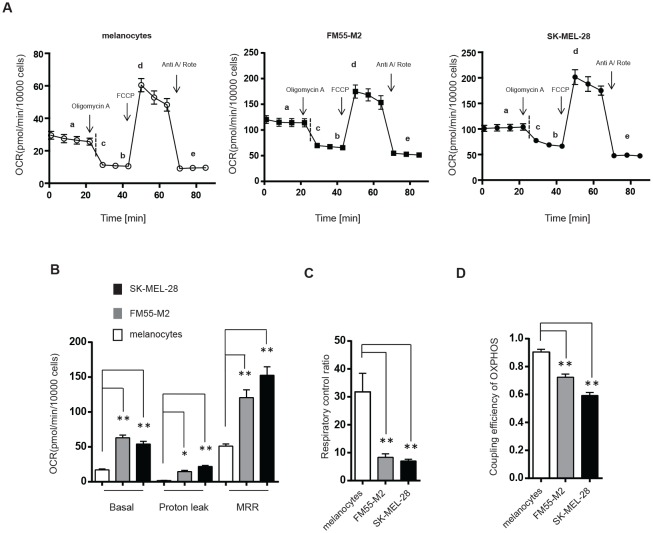
Mitochondria in melanoma cells consume oxygen but exhibit dysfunctional OXPHOS (A) OCR (pmol/min/10000 cells) in melanocytes and melanoma cells (FM55-M2 and SK-MEL-28) following sequential addition of oligomycin (1 μM), carbonylcyanide-p-trifluoromethoxyphenylhydrazone (FCCP) (optimized to 0.5 μM in FM55-M2 and SK-MEL-28 cells and 1.2 μM in melanocytes) and a mixture of antimycin A (Anti A; 2.5 μM) and rotenone (Rote; 2.5 μM). Different states of mitochondrial respiration are marked in the figure; basal respiration (a), proton leak (b), oligomycin sensitive respiration (c= a-b), maximal respiratory rate (MRR, d) and non-mitochondrial respiration (e). (B) Comparison of mitochondrial respiratory states (basal respiration, proton leak and MRR) in melanocytes and melanoma cells following corrections for non-mitochondrial respiration. (C) Calculation of the respiratory control ratio (RCR) (d/b) and (D) the coupling efficiency of OXPHOS (c/a) in melanocytes and melanoma cells. Data are presented as the means ± SD (n = 12) from one experiments and are representative of three independent experiments. Statistical analyses (B, C and D) were performed with one-way ANOVA, using Tuckey's multiple comparisons test to calculate significance (* = *p* < 0.05; ** = *p* < 0.01).

According to the “Warburg effect” paradigm, melanoma cells would be expected to take up glucose and convert it into lactate under standard (aerobic) cell culture conditions, whereas their normal cell counterpart, i.e. epidermal melanocytes, would convert glucose into pyruvate to be utilized by mitochondria. In order to confirm this basic metabolic difference, we made a series of real-time measurements of oxygen consumption rate (OCR) and extracellular acidification rate (ECAR) in human epidermal melanocytes and two melanoma cell lines, FM55-M2 and SK-MEL-28, using the Seahorse XF analyzer (Seahorse Bioscience). In agreement with the Warburg effect, we found that the OCR/ECAR ratio was much higher in melanocytes compared to melanoma cell lines, indicating that melanocytes are more dependent on OXPHOS whereas melanoma cells rely more on glycolysis, leading to a higher lactate production (Figure 1A). Hence, ECAR was significantly higher in melanoma cells compared to melanocytes (14-fold higher in FM55-M2 and 16-fold higher in SK-MEL-28) (Figure 1B). Using a cell respiratory control protocol, the XF analyzer also allowed us to discriminate individual respiratory states such as basal respiration, leak respiration and maximal respiration [40]. We found that all of these parameters were higher in melanoma cells compared to melanocytes (Figure 2A), even after adjusting for non-mitochondrial respiration (Figure 2B). The fact that melanoma cells exhibit a lower OCR/ECAR ratio than melanocytes is therefore not attributable to low oxygen consumption but rather reflects the high production of lactate in melanoma cells.

It is noteworthy that oxygen consumption *per se* says little of the efficiency of mitochondrial respiration. In fact, both melanoma cell lines showed considerably higher proton leak over the mitochondrial inner membrane than melanocytes (Figure 2B), which is a strong indicator of increased mitochondrial dyscoupling [40,41]. To obtain comparable estimates of OXPHOS efficiency in melanocytes and melanoma cells according to published guidelines [40], we subsequently calculated the mitochondrial respiratory control ratio (RCR) and the coupling efficiency of OXPHOS based on the analysis of mitochondrial respiratory states in Figure 2B. Compared to melanocytes, we found that both melanoma cell lines had significantly lower RCR values (Figure 2C) and a significantly lower coupling efficiency (Figure 2D). The RCR value signifies the capacity for oxidation of respiratory substrates and ATP synthesis, whereas the coupling efficiency denotes the number of protons being used by the ATP synthase for generation of ATP relative to the number of protons leaking over the mitochondrial inner membrane. Collectively, the higher proton leak *per se* as well as the lower values of respiratory control and coupling efficiency show that the OXPHOS in the two melanoma cell lines is dysfunctional compared to the OXPHOS in melanocytes.

### Melanoma cells depend on glycolysis for energy production

**Figure 3:**
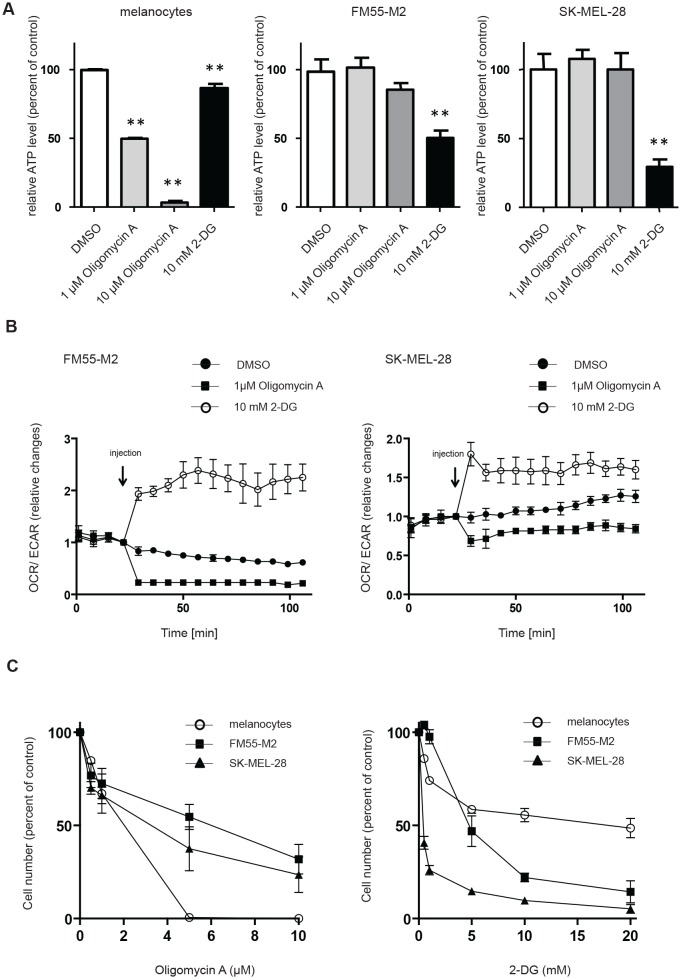
Melanocytes and melanoma cells depend on different metabolic pathways for energy production (A) ATP levels in melanocytes, FM55-M2 and SK-MEL-28 following 4 hours incubation with inhibitors of OXPHOS (oligomycin) or glycolysis (2-DG). Data are presented as relative changes compared to DMSO treated control and presented as the means ± SD (n = 5). Statistical analysis were performed with one-way ANOVA and Tuckey's multiple comparisons test (** = *p* < 0.01). (B) Changes in the OCR/ECAR ratios following additions of oligomycin (1 μM) or 2-DG (10 mM) to melanoma cells. Data are representative of two independent experiments. (C) Quantification of adhering melanocytes and melanoma cells following 96 hours incubation with a range of concentrations of oligomycin or 2-DG.

The dysfunctionality of OXPHOS in FM55-M2 and SK-MEL-28 cells suggests that these cells would be more dependent on glycolysis for ATP production than melanocytes. Indeed, treatment of melanoma cells with the hexokinase inhibitor 2-DG led to a prominent drop in ATP, whereas the mitochondrial ATPase inhibitor oligomycin A had limited effects (Figure 3A). Notably, the drop in ATP was observed in both FM55-M2 and SK-MEL-28 cells, even though these cells attempted to reverse to the use of OXPHOS when experiencing a block to glycolysis (Figure 3B). It was therefore evident that the mitochondria in these cells provided no useful alternative to the production of ATP in complete agreement with the fact that OXPHOS is dysfunctional. The reaction of melanoma cells was in striking contrast to the reaction of melanocytes, which exhibited high sensitivity to oligomycin A but a limited response to 2-DG (Figure 3A), indicating that melanocytes primarily derive ATP through mitochondrial ATP-synthase. Concurrently with its effects on ATP production in melanocytes, oligomycin A also limited the number of adhering melanocytes in culture at concentrations above 5 μM, whereas FM55-M2 and SK-MEL-28 cells were clearly less sensitive. Conversely, melanocytes were less sensitive to 2-DG than either of the melanoma cell lines (Figure 3C). Following treatment with 2-DG, cultures of melanoma cells exhibited prominent signs of cell detachment and cell death. Furthermore, the majority of the remaining adherent cells showed abnormal morphology and positivity for the senescence marker SA-β-gal (Supplementary Figures S1 and S2).

### The ^V600E^BRAF oncogene sustains melanoma cells via up-regulation of glycolytic and pentose phosphate pathway enzymes

**Figure 4:**
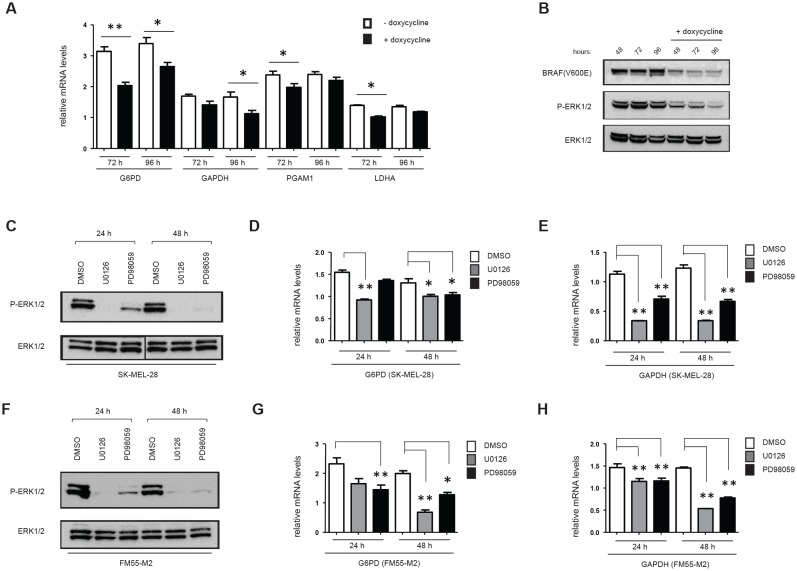
Expression of metabolic genes is affected by ^V600E^BRAF and MEK activity (A) G6PD, GAPDH, PGAM1 and LDHA mRNA levels in FM55-M2:^V600E^BRAF^sh^ cells were analyzed by real-time qPCR following 72 and 96 hours of^V600E^BRAF knock-down. Data are presented as the means ± SD for 3 parallel experiments and statistical analysis was performed with students t-test to calculate significance (n = 3) (* = *p* < 0.05; ** = *p* < 0.01). (B) Immunoblot analysis showing level of^V600E^BRAF and phospho-p44/42 MAPK (Erk1/2) (Thr202/Tyr204) following 48, 72 and 96 hours of^V600E^BRAF knock-down. (C -H) Immunoblot analysis of ERK1/2 activation (C and F) and analysis of G6PD and GAPDH mRNA levels (D, E, G and H) in melanoma cells treated with DMSO or MEK inhibitors (UO126 or PD98059, 10 μM each) for 24 or 48 hours. (C-E) SK-MEL-28 cells; (F-H) FM55-M2 cells. G6PD and GAPDH mRNA levels were analyzed by real-time qPCR. Data are based on three experiments for each gene and normalized to the expression of the housekeeping gene *RPLP0*, based on average from three experiments. Statistical analyses were performed with one-way ANOVA and Tuckey's multiple comparisons test (* = *p* < 0.05; ** = *p* < 0.01).

**Figure 5:**
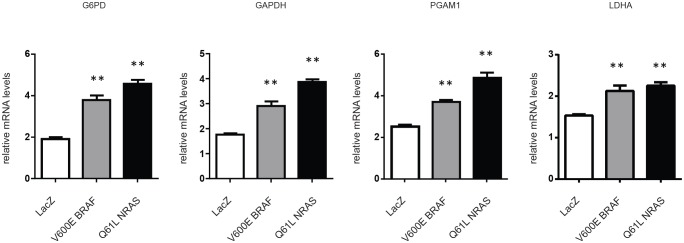
Ectopic expression of ^V600E^BRAF or ^Q61L^NRAS increases the expression of metabolic genes Expression of G6PD, GAPDH, PGAM1 and LDHA was investigated in melanocytes following transfection with plasmids expressing activated oncogenes (^V600E^BRAF or^Q61L^NRAS) or control plasmid (containing Lac-Z). The expression of the genes was normalized to the expression of the housekeeping gene RPLP0, based on average from three experiments. Data are presented as the means ± SD of three experiments and statistical analysis was performed using one-way ANOVA and Tuckey's multiple comparisons test (** = *p* < 0.01).

The morphological response of FM55-M2 cells to inhibition of glycolysis was similar to the response observed in these cells following knock-down of mutated BRAF [17]. This raised the question of whether loss of ^V600E^BRAF reduces the glycolytic process. Therefore, we generated an inducible system for knocking down ^V600E^BRAF in melanoma cells (FM55-M2:TREx: ^V600E^BRAF^sh^), allowing the examination of changes in gene expression following specific knock-down of ^V600E^BRAF induced by doxycycline treatment. Indeed, various glycolytic and PPP genes were significantly down-regulated following doxycycline treatment. Hence, after 72 hours the mRNA levels of glyceraldehyde-3-phosphate dehydrogenase (GAPDH), phosphoglyceromutase (PGAM1), lactate dehydrogenase (LDHA) and glucose-6-phosphate dehydrogenase (G6PD) were reduced in the range of 17-35% (Figure 4A). Minor but significant reductions in mRNA levels were also observed for triose phosphate isomerase (TPI1) and phosphogluconate dehydrogenase (PGD), whereas no significant changes were recorded for glucose-phosphate isomerase (GPI), aldolase (ALDOA) or gluconolactonase (PGLS) (data not shown). The observed reductions in glycolytic and PPP gene expression coincided with the reduced levels of ^V600E^BRAF and activated ERK (phospho-p42/44-MAPK) (Figure 4B). This trend persisted when MEK inhibitors were used as an alternative to RNA interference. Hence, the levels of GAPDH and G6PD mRNA followed the level of residual phospho-ERK in both SK-MEL-28 (Figure 4C, D, and E) and FM55-M2 (Figure 4F, G, and H) treated with U0126 or PD98059. To further confirm that BRAF along with other components of the RAS-BRAF-MEK-ERK signaling axis regulate glycolysis and PPP enzymes, melanocytes were transfected with plasmids encoding LacZ, ^V600E^BRAF or ^Q61L^NRAS followed by examination of mRNA levels. As shown in Figure 5, ^V600E^BRAF and ^Q61L^NRAS significantly increased the mRNA levels of G6PD, GAPDH, PGAM1 and LDHA to a similar extent.

### Impaired ^V600E^BRAF signaling leads to reversal of the Warburg effect

**Figure 6:**
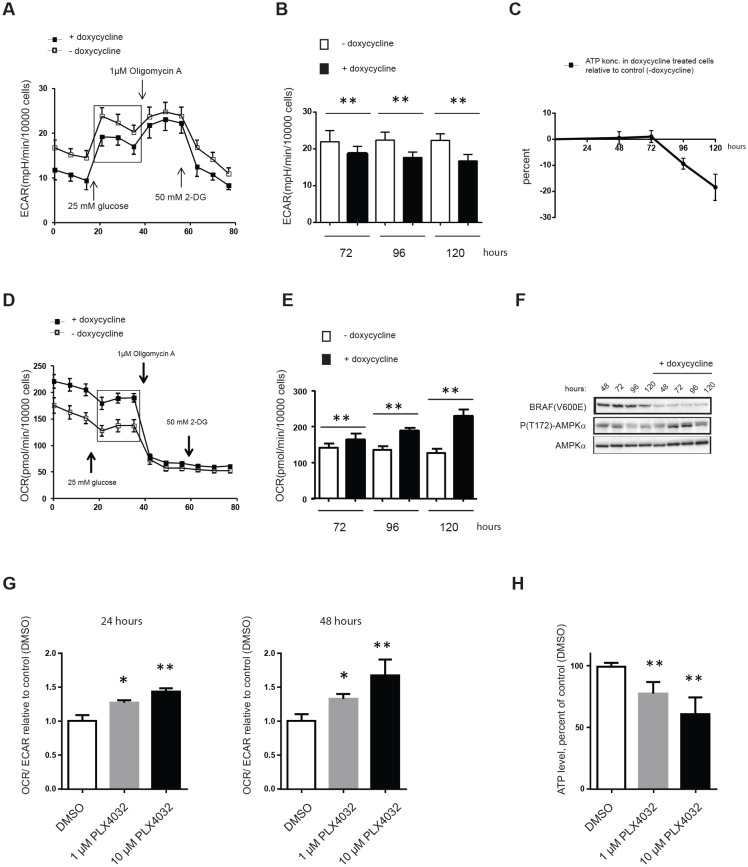
Reversal of the Warburg effect following ^V600E^BRAF knock-down results in ATP depletion OCR, ECAR and ATP levels in FM55-M2: TREx: ^V600E^BRAF^sh^cells undergoing doxycycline-induced knock-down of ^V600E^BRAF (A-F) or SK-MEL-28 cells undergoing PLX4032 mediated inhibition of ^V600E^BRAF (G and H). (A) Real-time measurements of ECAR in FM55-M2 following doxycyclin-induced knock-down of ^V600E^BRAF for 96 hours. (B) Basal ECAR following doxycyclin-induced knock-down of ^V600E^BRAF for 72, 96 or 120 hours. (C) Measurement of ATP levels following doxycyclin treatment for 48, 72, 96 and 120 hours. (D) Real-time measurements of OCR in FM55-M2 following doxycyclin-induced knock-down of ^V600E^BRAF for 96 hours. (E) Basal OCR values following doxycyclin-induced knock-down of ^V600E^BRAF for 72, 96 or 120 hours. (F) Immunoblot analysis of FM55-M2: TREx: ^V600E^BRAF^sh^cells undergoing doxycycline-induced knock-down of ^V600E^BRAF. Shown are the levels of ^V600E^BRAF, phospho-(Thr172)-AMPKα and total AMPKα at different time-points. (G) Real-time measurements of OCR/ECAR ratios in SK-MEL-28 following treatment with DMSO or PLX4032 (1μM or 10μM) for 24 or 48 hours. Data are presented as the means ± SD (n = 6) from one experiment and are representative of two independent experiments. (H) ATP levels in SK-MEL-28 following treatment with 1 or 10 μM of PLX4032 for 48 hours. Data were normalized for protein content and is presented as the means ± SD (n = 8). Statistical analyses were performed with one-way ANOVA and Tuckey's multiple comparisons test (* = *p* < 0.05; ** = *p* < 0.01).

Since suppression of ^V600E^BRAF signaling was found to reduce, albeit moderately, the expression of several glycolytic and PPP genes in melanoma, it became of interest to investigate whether these changes in combination affected the rate of glycolysis (ECAR). Indeed, we found that ^V600E^BRAF knock-down reduced ECAR by 15% after 72 hours and 25% after 120 hours (Figure 6A and 6B). Notably, as seen also when using 2-DG (Figure 3B), reduction in ECAR was accompanied by increased OCR indicating an attempt by melanoma cells to compensate for even minor reductions in glycolytic activity by upregulating OXPHOS (Figure 6D and 6E). In fact, following 120 hours of ^V600E^BRAF knock-down, FM55-M2 cells had nearly doubled their OCR (Figure 6E). However, as also seen after treatment with 2-DG (Figure 3A), the increased OXPHOS was unable to compensate for loss of energy produced by ECAR and ATP levels were found to gradually decline following ^V600E^BRAF knock-down (Figure 6C). The AMP kinase (AMPK) is a known sensor of bioenergetic stress, which is activated by increased levels of AMP but at the same time is under negative control of the BRAF-MEK-ERK signaling cascade [42]. As expected, we found that the combination of both reduced ATP and ^V600E^BRAF levels caused activation of AMPK as seen from increased phosphorylation at Thr172 of the alpha subunit of AMPK in doxycycline treated samples (Figure 6F). This indicates that FM55-M2 cells undergoing a reversed Warburg effect are confronted with the inability of their dysfunctional OXPHOS to compensate for loss of glycolytic ATP production and subsequently they experience bioenergetic stress.

A metabolic shift characterized by reduced glycolysis and increased OXPHOS was also observed in SK-MEL-28 cells treated with the specific ^V600E^BRAF inhibitor vemurafenib/PLX4032. Hence, incubation with 10 μM PLX4032 produced OCR/ECAR ratios that were 43% and 68% higher than the control after 24 and 48 hours, respectively (Figure 6G). However, incubation with PLX4032 for 48 hours also reduced ATP levels in SK-MEL-28 (Figure 6H). These data mimic the results obtained with FM55-M2 cells using knock-down of ^V600E^BRAF and confirm that the mitochondria in SK-MEL-28 cells are also dysfunctional with respect to OXPHOS.

### Overexpression of GAPDH abrogates oncogene addiction

**Figure 7:**
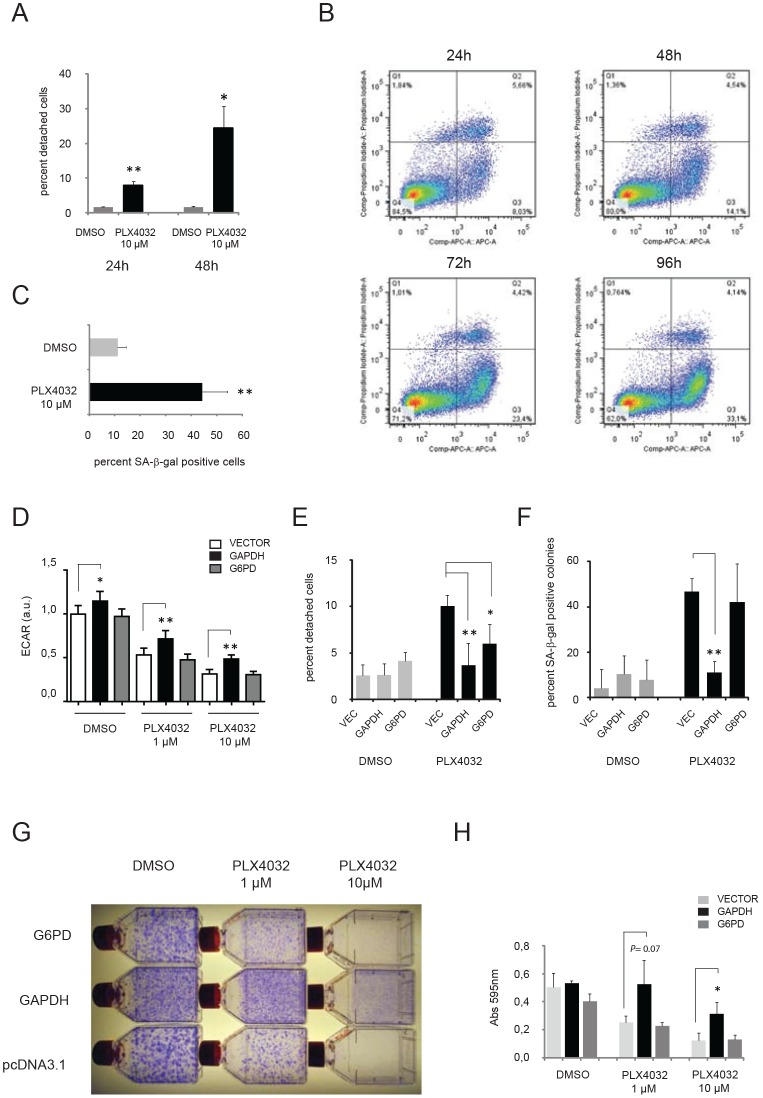
Overexpression of GAPDH abrogates oncogene addiction Examination of SK-MEL-28 cells treated with PLX4032 (A) percentage of cells detaching following treatment with DMSO or 10μM PLX4032 for 24 or 48 hours. (B) FACS analysis of Annexin V-stained cells revealing the percentage of apoptotic cells at different time-point following treatment with 10μM PLX4032 (C) percentage of cells showing positivity for senescence-associated beta-galactosidase (SA-β-gal) following treatment with 10 μM for one week. (D-H) The effect of stable overexpression of metabolic enzymes in SK-MEL-28 cells prior to treatment with PLX4032. Cells were transfected with plasmids conferring resistance to G418 and having either no insert (vector) or expressing G6PD or GAPDH. Selection with G418 was performed for 14 days before treatment with PLX4032. (D) Extracellular acidification rates in stably transfected cultures treated for 48 hours with DMSO or PLX4032 (1μM or 10μM). (E) Percentage of cells in stably transfected cultures detaching following treatment for 48h with DMSO or 10μM PLX4032. (F) Percentage of G418-resistant colonies showing positivity for SA-β-gal following treatment with 1 μM for one week. (G) Representative pictures from a colony formation assay involving selection with G418 for two weeks followed by treatment with DMSO or PLX4032 (1μM and 10μM). (H) Quantification of adhering cells in (G) by crystal violet staining, extraction of the dye and measurement of A595. Shown are the means ± SD of three independent experiments. Unless stated otherwise, all experiments shown are representative of two independent experiments

The SK-MEL-28 cell line represents a useful model of oncogene addiction, since we and others have shown that this cell line responds to MEK inhibitors or knock-down of ^V600E^BRAF. In agreement with these previous findings we found that treatment with PLX4032 caused a mixture of different changes in SK-MEL-28 cultures, including increased cell detachment (Figure 7A), increased apoptosis (Figure 7B), and increased positivity for the senescence marker SA- *β*-gal in adherent cells (Figure 7C). To investigate whether metabolic changes represent the mechanistic basis for these cellular changes, we stably expressed GAPDH or G6PD in SK-MEL-28 cells and subjected these cells to treatment with PLX4032, looking in parallel for rescue of glycolysis and cell fate. Overexpression of GAPDH indeed counteracted the loss of ECAR otherwise seen upon administration of PLX4032 (Figure 7D), and greatly reduced the number of cells detaching or undergoing senescence due to PLX4032 (Figure 7E and F). In fact, overexpression of GAPDH caused an overall increase in the number of adherent cells stained by crystal violet subsequent to treatment with 10 μM PLX4032 for a week (Figure 7G and H). In contrast, overexpression of G6PD showed no rescue of senescence (Figure 7F), and although it counteracted cell detachment induced by PLX4032 (Figure 7E), it was unable to cause an overall increase in the number of adherent cells (Figure 7G and H). These data demonstrate the causal interconnections between loss of ^V600E^BRAF activity, metabolic deficiency and cellular fate, and provide direct evidence that oncogene addiction rests on a metabolic foundation in a known melanoma model.

## DISCUSSION

Several studies indicate that melanoma cells are addicted to constitutive activation of the RAS-RAF-MEK-ERK cascade. Treatment with vemurafenib or trametinib has shown remarkable effects in melanoma patients [21,26] and knock-down or inhibition of ^V600E^BRAF leads to growth arrest, senescence or apoptosis in various melanoma models *in vitro* [15-19]. We have previously used FM55-M2 and SK-MEL-28 cells as model systems to show that RNAi-mediated knock-down of ^V600E^BRAF leads to a mixture of growth arrest, loss of adherence, senescence and apoptosis [17]. Interestingly, both cell lines carry mutations in *TP53* as well as the RB pathway [43-45], suggesting that other mechanisms exist in these melanoma cells that potentially overrule the inactivation of key tumor suppressor pathways and enforce senescence or cell death. In this respect, FM55-M2 and SK-MEL-28 cells exhibit *bona fide* oncogene addiction including all the phenotypical hallmarks, and also the mechanistic unknowns presently associated with this phenomenon [1].

Further to the previous studies of FM55-M2 and SK-MEL-28 we now provide evidence for a metabolic rationale behind the addiction to ^V600E^BRAF. Firstly, both melanoma cell lines display a striking addiction to glycolysis due to underlying OXPHOS dysfunction. Secondly, ^V600E^BRAF upholds the activity of glycolysis and therefore the addiction to glycolysis *de facto* becomes an addiction to ^V600E^BRAF. Thirdly, the senescence response associated with inhibition of ^V600E^BRAF is rescued by overexpression of GAPDH providing direct evidence that oncogene addiction has a metabolic foundation.

It is almost nine decades ago that Otto Warburg first described how cancer cells continue to metabolize sugar in the presence of oxygen [33], and it is now generally accepted that cancer cells exhibit a higher uptake of glucose and derive a greater fraction of their ATP from glycolysis rather than from OXPHOS. Notably, metastatic melanomas are well detected by positron emission tomography (PET) on the basis of their avid uptake of 18F-FDG [46], and a glycolytic phenotype was recently associated with malignant melanoma cell lines by metabolic profiling [47]. These findings are in agreement with the basic metabolic characteristics observed in the two melanoma cell lines used in our study. Also, the influence on glycolytic activity exerted by ^V600E^BRAF has been indicated by previous findings. Hence, an effect of PLX4032 was recently demonstrated on the uptake of 18F-FDG in melanoma cells, whereas inducible expression of ^V600E^BRAF increased the glucose uptake rate and decreased O_2_ consumption in thyroid cancer cells [38,39]. In agreement with the present findings, metabolic control by ^V600E^BRAF could be envisaged on the basis of its signaling through the MEK and ERK kinases and activation of MYC or HIF-1α, both of which are major determinants of cancer metabolism [37,48].

Discrepancy between present and previous findings, however, exists when one considers the underlying reasons for the addiction of cancer cells to glycolysis, which we claim here to be OXPHOS dysfunction. Following his pioneering observations, Otto Warburg actually proposed that mitochondrial dysfunction could constitute the underlying reason for the increased glycolytic activity [49], and indeed, there are interesting examples of mutations in cancer cells that specifically affect mitochondrial functions [50-52]. However, such mutations are rare when looking at cancers as a whole and other work has shown that mitochondria in cancer cells continue to consume oxygen at rates comparable to or even higher than normal mitochondria [53]. Mitochondria in cancer cells also remain capable of adapting to changes in substrate availability [54] and may even run the tricarboxylic acid cycle (TCA) in the reverse direction [47]. Therefore, the old concept of mitochondrial dysfunction in cancer has gradually been displaced by a number of theories commonly stating that the Warburg effect exists because it constitutes a selective growth advantage to cancer cells [55]. Hence, an increased rate of glycolysis allows for growth under hypoxic conditions, boosts anti-oxidant defense through NADPH production in the pentose phosphate shunt, and through the production of lactate contributes to an acidic microenvironment which is obstructive to stromal cells [29,31,32,49]. Also, it has been proposed that proliferating cells (such as cancer cells) use a higher glycolytic rate to allow biosynthesis of macromolecules through various anabolic pathways that exist as branches of the glycolytic process [29,31].

Despite the old concept of mitochondrial dysfunction in cancer has been drawn into question [29,31,32,49], compelling reasons exist why especially OXPHOS dysfunction should still be considered. Technological advances in the form of the Seahorse XF analyzer has made it possible to conduct real-time, parallel measurements of glycolysis and OXPHOS in viable cells, which has greatly facilitated studies of mitochondria when the Warburg effect is reversed. Hence, using Seahorse technology, lung cancer cell lines A549 and H460 have been shown to switch back to OXPHOS when treated with the glycolysis inhibitor 2-DG [56]; and using the same technique we have now shown a similar switch in two melanoma cell lines after treatment with 2-DG as well as after knock-down of ^V600E^BRAF or treatment with vemurafenib. Such “reversed Warburg effect” conditions liberate the mitochondria from the proposed suppression exerted by high glycolytic activity (commonly known as the Crabtree effect) [57] and would readily disclose any mitochondrial dysfunction if it exists. In agreement with the previous work on lung cancer cell lines [56], we found that the mitochondria are unable to uphold ATP production during reversal of the Warburg effect and hence demonstrate that the mitochondria are dysfunctional with respect to OXPHOS. This dysfunction was supported by a more detailed analysis of cellular respiratory control. Compared to melanocytes, both melanoma cell lines exhibited considerably lower coupling efficiencies and respiratory-control-ratios, which signifies poor capacity for oxidation of respiratory substrates and ATP synthesis or a high degree of proton leak [40].

It is noteworthy that OXPHOS dysfunction would not have been seen based only on overall oxygen consumption rates. In fact, both melanoma cell lines exhibited OCR values higher than melanocytes; and in parallel experiments we also observed higher citrate synthase activity in both cancer cell lines compared to melanocytes (data not shown). *Per se*, this indicates that the mitochondria are not inactive in the two melanoma cell lines, and it illustrates the importance of performing more detailed analysis before conclusions can be reached on mitochondrial dysfunction. Notably, enzymes of the TCA cycle may be found at higher levels or more active in cancer cells due to increased glutamine oxidation (glutaminolysis) [47,58]. General statements saying that mitochondria are dysfunctional in cancer cells are not warranted based on the present or previous studies; and we merely claim that OXPHOS is dysfunctional in two malignant melanoma cell lines explaining their addiction to ^V600E^BRAF-driven glycolysis and hence oncogene-addiction.

Recently, other potential explanations for oncogene addiction have emerged with respect to ^V600E^BRAF. Lee et al. (2011) reported that ^V600E^BRAF may actively suppress mitochondrial oxidative phosphorylation in thyroid cancer cells in an ERK-independent manner [39]. We have found no evidence of ^V600E^BRAF -mediated suppression of OXPHOS in melanoma cells since 2-DG induced a very efficient reversal of the Warburg effect despite the endogenous presence of ^V600E^BRAF in both FM55-M2 and SK-MEL-28 cells. The metabolic shift therefore appears to be effectuated in response to energy deficiency, only subject to indirect regulation by ^V600E^BRAF through its effects on the levels of glycolytic enzymes. Furthermore, and contrary to the work of Lee et al. (2011), we found that both metabolic and phenotypical changes resulting from knock-down of ^V600E^BRAF could be mimicked by MEK inhibitors.

Acquisition of resistance to PLX4032 is a major problem in the clinical treatment of melanoma [27,28]. In this respect, recent work showed that resistance to PLX4720 (the tool compound for PLX4032) is associated with reactivation of ERK1/2 signaling, and suppression of the pro-apoptotic B-cell leukemia/lymphoma 2 (Bcl-2) homology domain 3 (BH3)-only proteins [59]. Based on these data it may be speculated that the RAS-BRAF-MEK-ERK pathway exerts a general suppression of apoptosis providing a possible explanation for the addiction of melanoma cells to ^V600E^BRAF. Although we did observe increased levels of apoptosis in SK-MEL-28 after 3 days of treatment with PLX4032, and in FM55-M2 after knock-down of ^V600E^BRAF [17], our data indicate that one of the primary effects of reducing BRAF activity is cellular detachment, which can be observed within 24 hours of PLX4032 treatment. Cellular detachment is a known inducer of apoptosis by itself (anoikis) and apoptosis is intimately linked to the metabolic state of a cell [60,61]. It is therefore unclear whether induction of apoptosis following treatment with PLX4032 represents a direct effect of relieving an alleged suppressive effect of ^V600E^BRAF or rather an indirect effect of cell detachment or energy deficiency. Here, we have shown that overexpression of GAPDH is capable of rescuing SK-MEL-28 cells from the detrimental effects of PLX4032 including cell detachment and senescence amongst adherent cells. These data support the view that metabolic deficiency is of central importance to at least some of the cell fate decisions provoked by inhibition of ^V600E^BRAF.

In summary, we have provided a detailed account of the metabolic changes resulting from inhibition or knock-down of ^V600E^BRAF in two malignant melanoma cell lines. Our data provide new experimental support for OXPHOS dysfunction in cancer cells and argue that oncogene addiction rests on a metabolic foundation. Oncogene addiction is the basis for novel cancer treatment targeting individual kinases and pathways activated by oncogenic mutations but the biochemical basis for its efficacy has remained elusive. The fact that cancer cells are addicted to oncogene-driven energy production due to OXPHOS dysfunction suggests that existing cancer therapy may be improved by compounds aimed at glycolysis or alternative routes of ATP production.

## MATERIALS AND METHODS

### Cell culture

The melanoma cell lines (FM55-M2 and SK-MEL-28) were cultured in RPMI 1640 medium, supplemented with 10% (v/v) fetal bovine serum and 0.1% (v/v) penicillin-streptomycin. The inducible cell line FM55-M2: TREx: ^V600E^BRAF^sh^ was likewise cultured in RPMI 1640 medium, but supplemented with 10% (v/v) tetracycline system approved fetal bovine serum (Clontech). Melanocytes were cultured in Medium 254 for melanocytes with Human Melanocyte Growth Supplement, 0.1% (v/v) penicillin-streptomycin and 0.1% (v/v) gentamicin-amphomycin. All cell cultures were maintained in atmospheric conditions at 37°C of 5% CO_2_ and 21% O_2_.

### Analysis of oxygen consumption rate (OCR) and glycolytic rate (ECAR) in intact cells

Investigation of OCR/ECAR ratios and mitochondrial function in melanoma cells (FM55-M2 and SK-MEL-28) and melanocytes were performed using XF96 or XF24 analyzers with XF96 V3 or XF24 V7 cell culture microplates, respectively (Seahorse Bioscience). When measurements were performed after 24 hours, 2 × 10^4^ melanoma cells/well or 3 × 10^4^ melanocytes/well were seeded in XF96 V3 microplates; or 3 × 10^4^ melanoma cells/well were seeded in XF24 V7 microplates. When measurements were performed during several days, melanoma cells were seeded in XF24 V7 cell culture microplates at 1 × 10^4^ cells/well in growth medium the day before. OCR and ECAR were investigated in Seahorse assay buffer (containing 10 mM glucose, 10 mM pyruvate, pH 7.4) or basic glucose free DMEM medium (pH 7.4). Mitochondrial respiratory states, respiratory control ratio (RCR) and coupling efficiency of OXPHOS were investigated according to previously published protocol [40]. The following compounds and concentrations were added dependent on type of experiment: glucose (25 mM); oligomycin A (1 μM); FCCP (optimized concentrations; 0.5 μM for melanoma cells and 1.2 μM for melanocytes); antimycin A (2.5 μM); rotenone (2.5 μM); 2-deoxyglucose (10 mM or 50 mM) and PLX4032 (1 μM or 10 μM). When measurements were performed during several days, melanoma cells were seeded and grown in parallel XF24 V7 microplates to allow determination of cell number at different time points by crystal violet staining as described below.

### ATP measurements

The concentration of ATP was determined using a modified method based on the ATPlite luminescence assay system (Perkin Elmer). Depending on type of experiment, either 1.0 or 2.5 × 10^5^ cells were seeded in 5 cm dishes. Samples were collected by lysing the cells with 750 μL lysis solution (2:1 RPMI-1640 medium: cell lysis buffer (ATPlite, Perkin Elmer)) and immediately frozen in liquid nitrogen. For every measurement triplicate samples were made. Luminescence was recorded in a Fluostar Omega microplate reader (BMG labtech), and ATP levels normalized for protein content determined using Bradford assay or cell size based on parallel measurements using a Z2 Coulter particle and cell counter (Beckman Coulter).

### Generation of FM55-M2: T-REx: ^V600E^BRAFsh

A plasmid capable of shRNA expression from a doxycyclin-inducible promoter was generated on the basis of a previously described pSuper: G418^R^ vector [17]. In brief, tetracycline operons were placed on either side of the TATAA box in the H1 promoter of the pSuper vector: G418^R^; and the following oligoes5'-gatccccGCTACAGAGAAATCTCGATttcaagagaATCGAGATTTCTCTGTAGCtttttggaaa-3' 5'-agcttttccaaaaaGCTACAGAGAAATCTCGAT tctcttgaaATCGAGATTTCTCTGTAGCggg-3' were annealed and cloned in the *Bgl* II and *Hind* III sites of pSUPER/G418^R^ to generate the pSuper: G418^R^: ^V600E^BRAF^sh^ plasmid that produces shRNA targeting BRAF mRNA with the c.1799T>A; p.V600E mutation. A clonal line of FM55-M2 was engineered to stably express the tetracycline repressor protein from pcDNA6:T-REx plasmid (Invitrogen, Carlsbad, CA) and subsequently transfected with pSuper: G418^R^: ^V600E^BRAF^sh^ plasmid. A stable clone was chosen on the basis of its ability to show knock-down of ^V600E^BRAF upon administration of 1 μM doxycyclin.

### Cloning of ^Q61L^- NRAS expression vector

NRAS carrying the c.182A>T (p.Q61L) mutation was cloned from the human malignant melanoma cell line FM79 [45]. In brief, total RNA was isolated using the RNeasy kit (Qiagen, Valencia, CA) and subjected to reverse transcription using the SuperScript III reverse transcriptase and hexamer primers (Invitrogen, Carlsbad, CA). cDNA corresponding to the entire open reading frame of NRAS was amplified using the Platinum Taq polymerase High Fidelity (Invitrogen, Carlsbad, CA) and the primers 5'-AAGCTTGCAGTGGAGCTTGAGGTTCTTG-3' and 5'-CTCGAGCCAGGGTGTCAGTGCAGCTT-3' thereby introducing HindIII and XhoI restriction sites (underlined) subsequently used for cloning into the pcDNA 3.1/Hygro^(+)^ plasmid (Invitrogen, Carlsbad, CA). The nucleotide sequence of the insert was verified by sequencing.

### Cell attachment/ detachment

In some experiments cellular attachment/detachment was quantified by parallel counting of cells floating in the medium prior to trypsinization and cells released after trypsinization. In other experiments, adherent cells were quantified using staining with crystal violet. In brief, cells were washed in PBS and fixed with glutaraldehyde for 15 minutes. The fixed cells were incubated with crystal violet solution (0.1% (w/v) crystal violet, 20% (v/v) CH_3_OH) for 1 hour. Subsequently, the dye was extracted by 10% (v/v) acetic acid for at least 18 hours and A595 recorded.

### Transfection of melanocytes

Transfection of human primary melanocytes was performed using Amaxa nucleofection technology. Buffer and program were as recommended by the manufacturer. Per reaction, 10^6^ cells and 5 μg of plasmids encoding LacZ, ^V600E^BRAF or ^Q61L^NRAS oncogene were suspended in 100 μL of Nucleofector Solution (Amaxa) along with 0.5 μg plasmid expressing GFP (amaxa). The transfected cells were seeded in fresh culture medium immediately after transfection and this medium was changed 4 hours after the transfection to remove dead cells and debris. Cells were collected after 24 hours and used for real-time qPCR analysis. Data were corrected for differences in transfection efficiency based on GFP expression.

### Quantitative RT-PCR

Gene expression levels were determined with real-time RT-PCR. RNA was isolated using NucleoSpin RNA II kit (Macherey-Nagel) and procedures were carried out as recommended by the manufacturer. The RNA concentration was measured in a 2100 Bioanalyzer (Agilent technologies) using the RNA 6000 Nano LabChip kit (Agilent technologies). cDNA was synthesized using the SuperScript III Reverse Transcriptase kit and hexamers-pentadecamer primers (TAG Copenhagen A/S), oligo dT_24_ (TAG Copenhagen A/S) and dNTPs (GE healthcare). Real-time qPCR was carried out using the LigthCycler FastStart DNA Master^PLUS^ SYBR Green I kit (Roche). The expression of the genes was normalized with respect to expression of the housekeeping gene *RPLP0*. Primers for quantitative RT-PCR analysis (Sigma-Aldrich) are listed in Table S1.

### Western blotting

Lysates were produced by scraping off the cells in the presence of hypotonic extraction buffer (20 mM Tris pH 7.6, 10 mM KCl, 2 mM MgCl_2_ and 1 mM EDTA) or RIPA buffer (250 mM NaCl, 1% NP-40, 0.5% sodium deoxycholate, 0.5% SDS and 50 mM Tris pH 8) supplemented with a protease inhibitor cocktail (Complete^TM^) and phosphatase inhibitors (PhosSTOP^TM^). The protein content was measured using the Coomassie Plus Protein Assay Reagent (Pierce), and 50-100 μg protein was separated on NuPAGE 4–12% Bis-Tris polyacrylamide gels (Invitrogen). Antibodies were as follows: rabbit anti-phosphorylated p44/p42 (P-Thr202/Tyr204) MAPK (Cell signaling #9101); rabbit anti- nonphosphorylated p44/p42 MAPK (Cell signaling #9102) and mouse anti- ^V600E^BRAF (Spring Bioscience, VE1).

### Assays for senescence and apoptosis

Senescent cells were detected using the senescence-associated β-galactosidase staining kit and the protocol recommended by the manufacturer (Cell Signaling Technology). Apoptosis was detected using APC Annexin V staining according to recommendations by the manufacturer (BD bioscience)

## Supplementary Figures and Tables




