# Supplementary material for: Dysfunctional oxidative phosphorylation makes malignant melanoma cells addicted to glycolysis driven by the V600EBRAF oncogene
Source: Oncotarget. 2013 Apr 8;4(4):584–99. doi: 10.18632/oncotarget.965 (PMC3720606; doi:10.18632/oncotarget.965)
Supplement: Supplementary file 1 [file oncotarget-04-584-s001.pdf]

Dysfunctional oxidative phosphorylation makes malignant melanoma cells addicted to glycolysis driven by the ^{V600E}BRAF oncogene - Hall et al

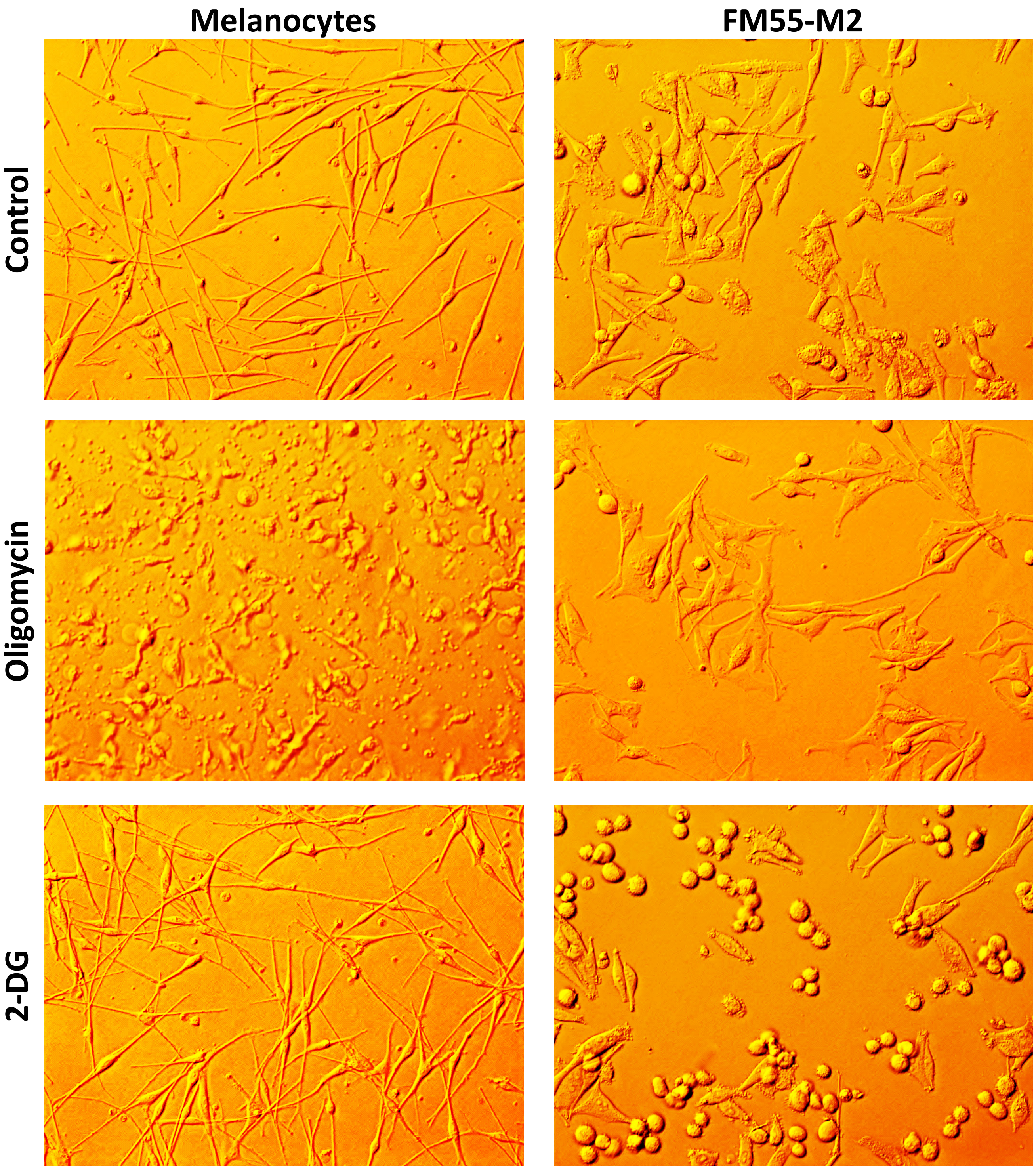

Figure S1: Morphological changes resulting from treatment with oligomycin A or 2-DG. Primary melanocytes and FM55-M2 melanoma cells were exposed to Oligomycin A (10 µM) or 2-DG (10 mM) for 24 hours.

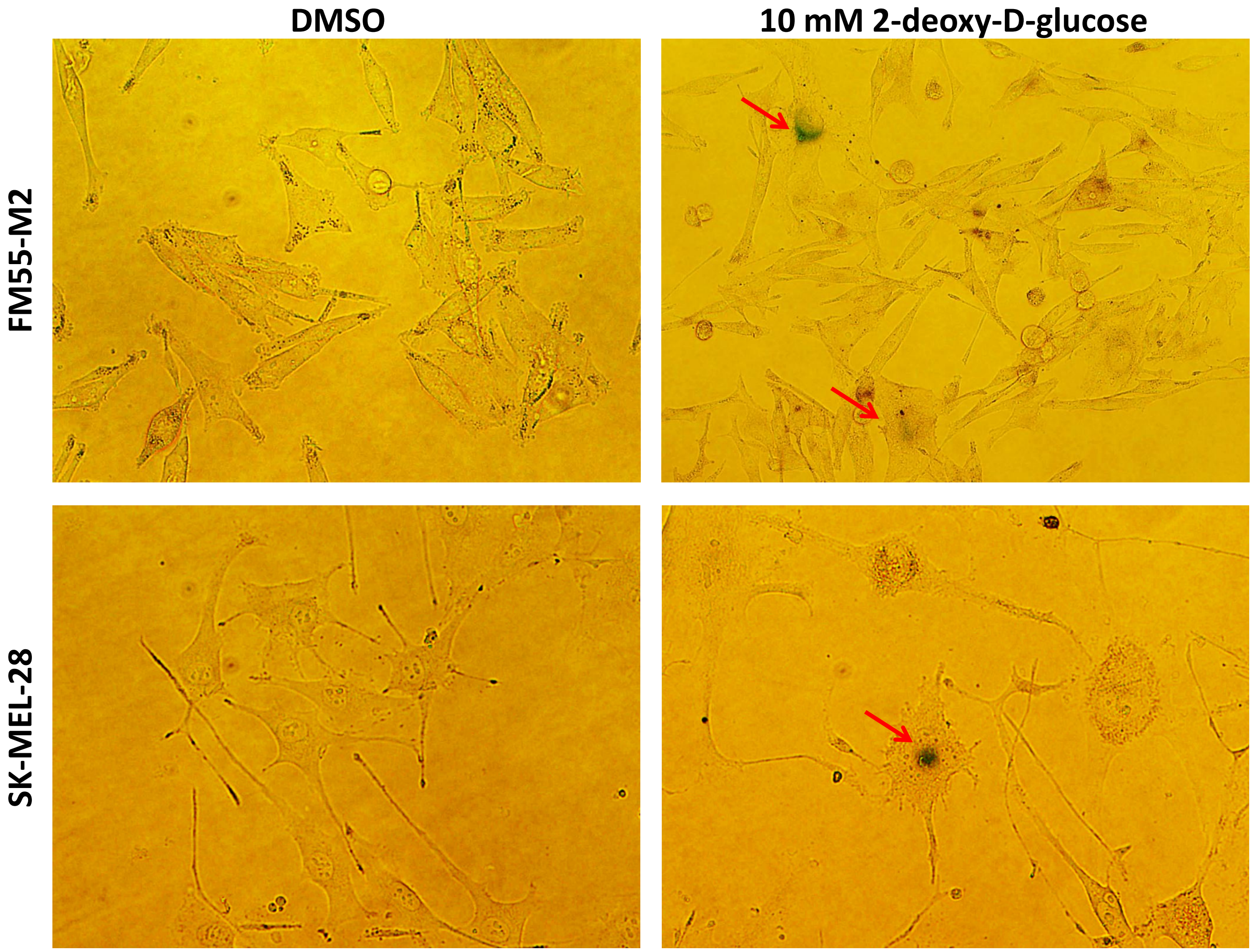

Figure S2: Changes to the morphology and SA-β-gal staining of melanoma cells treated with 2-DG. FM55-M2 and SK-MEL-28 malignant melanoma cells were treated with DMSO or 2-DG (10 mM) for one week. Cells remaining adherent were stained for SA-β-gal. At the onset of the experiment, cells to be treated with DMSO were deliberately plated at lower density in order to prevent the cultures from being too dense at the time of staining.
