# Supplementary material for: Dysfunctional oxidative phosphorylation makes malignant melanoma cells addicted to glycolysis driven by the V600EBRAF oncogene
Source: Oncotarget. 2013 Apr 8;4(4):584–99. doi: 10.18632/oncotarget.965 (PMC3720606; doi:10.18632/oncotarget.965)
Supplement: Supplementary file 2 [file oncotarget-04-584-s002.docx]

Dysfunctional oxidative phosphorylation makes malignant melanoma cells addicted to glycolysis driven by the **V600E**BRAF oncogene – Hall et al

**Table S1: Primer sequences**

| **Gene** | **Primer sequences (forward and reverse)**  **(5'→3')** | **cDNA number** | **Localisation** | **Annealing**  **Temperature (°C)** | **Product size (bp)** |
| --- | --- | --- | --- | --- | --- |
| *GAPDH* | *F:* AAATCCCATCACCATCTTCCA  *R:* AAATGAGCCCCAGCCTTCT | NM_002046 | F: exon 4  R: exon 5 & 6 | F: 53.1  R: 52.8 | 123 |
| *G6PD* | *F:* GCCAACCGCCTCTTCTACCT  *R:* GATGATGCGGTTCCAGCCTA | NM_000402 | F: exon 5  R: exon 5 & 6 | F: 54.8  R: 54.9 | 105 |
| *PGD* | *F:* TTATTTGTGGGGAGCGGAGT  *R:* CAGCAGGGTTCTCCAGTTCC | NM_002631 | F: exon 5  R: exon 6 | F: 53.6  R: 53.5 | 149 |
| *PGAM1* | *F:* TGTCAAGCATCTGGAGGGTCT  *R:* ACCGTCTCTTCATCCCCCA | NM_002629 | F: exon 3 & 4  R: exon 4 | F: 53.5  R: 53.9 | 138 |
| *TPI1* | *F:* TCAGAGCACCCGTATCATTTAT  *R:* GGCTCATTGTTTGGCATTG | NM_000365 | F: exon 6  R: exon 7 | F: 50.8  R: 51.3 | 148 |
| *RPLP0* | *F:* ACTAAAATCTCCAGGGGCACC  *R:* ATGACCAGCCCAAAGGAGAA | NM_053275 | F: exon 5  R: exon 6 | F: 53.7  R: 53.5 | 134 |
| *GPI* | *F:* GATGCCAGAGGTCAACAAGGT  *R:* CGTGATGGTCTTGCCTGTGTA | NM_000175 | F: exon 4  R: exon 5 | F: 53.0  R: 53.4 | 97 |
| *LDHA* | *F:* ATTGAAGGGAGAGATGATGGAT  *R:* AGCCGTGATAATGACCAGC | NM_005566 | F: exon 3  R: exon 4 | F: 50.8  R: 49.9 | 121 |
| *ALDOA* | *F:* TGGGCATCAAGGTAGACAAGG  *R:* TTCAGCACACAACGCCACTT | NM_184043 | F: exon 3 & 4  R: exon 5 | F: 54.1  R: 54.0 | 145 |
| *PGLS* | *F:* GCTGAGGACTACGCCAAGAAG  *R:* CAATCTTCTCCCGCTCCTGTA | NM_012088 | F: exon 2  R: exon 3 & 4 | F: 53.3  R: 53.5 | 148 |
